# Accumulation Characteristics of Natural *Ophiocordyceps sinensis* Metabolites Driven by Environmental Factors

**DOI:** 10.3390/metabo14080414

**Published:** 2024-07-27

**Authors:** Tao Wang, Chuyu Tang, Jianbo Chen, Jing Liang, Yuling Li, Xiuzhang Li

**Affiliations:** 1 State Key Laboratory of Plateau Ecology and Agriculture, Qinghai Academy of Animal Science and Veterinary Medicine, Qinghai University, Xining 810016, China; 13085500761@163.com (T.W.); chuyutang0410@163.com (C.T.); jianbochen0211@163.com (J.C.); 2 Ningxia Zhihui Yuanshi Winery Co., Yinchuan 750000, China; lj2391761290@163.com; 3 State Key Laboratory of Plateau Ecology and Agriculture, Qinghai Academy of Animal and Veterinary Science, Xining 810016, China

**Keywords:** *Ophiocordyceps sinensis*, metabolomics, redundancy analysis, metabolite response

## Abstract

The environment is an important factor affecting the composition and abundance of metabolites in *O. sinensis*, which indirectly determines its edible function and medicinal potential. This study integrated metabolomics and redundancy analysis (RDA) to analyze the metabolite profile characteristics and key environmental factors influencing *O. sinensis* in various production areas. A total of 700 differentially accumulated metabolites (DAMs) were identified, primarily comprising lipids, organic acids, and organoheterocyclic compounds. Results from hierarchical cluster analysis and KEGG indicated distinct accumulation patterns of these DAMs in *O. sinensis* from different regions, with enrichment in pathways such as tryptophan metabolism and glycerophospholipid metabolism. Environmental factors like annual mean precipitation, pH, temperature, and altitude were found to significantly influence metabolite composition, particularly lipids, organic acids, and nucleosides. Overall, this study highlights the impact of environmental factors on metabolite diversity in *O. sinensis* and sheds light on the evolutionary processes shaping its metabolic landscape.

## 1. Introduction

*Ophiocordyceps sinensis* (natural *O. sinensis* in this study) is a precious biological resource that is widely used in the fields of medical treatment, nutrition, and health care because of its rich variety of bioactive components that are beneficial to the human body [1]. *O. sinensis* has been found in many countries in East Asia, North America, and Southeast Asia. China is the world’s most abundant *O. sinensis* resource and is mainly distributed near the Qinghai–Tibet Plateau [2,3]. In China, *O. sinensis* reserves are distributed in a stepwise manner with an increase in altitude. *O. sinensis* has not been reported in low-altitude areas below 2000 m; in the high-altitude area of 2000 m–3000 m, *O. sinensis* is mainly distributed in Maqu County, Luqu County and Tianzhu Tibetan Autonomous County of Gansu Province, Xiaojin County of Sichuan Province, and Diqing Tibetan Autonomous Prefecture of Yunnan Province. In extremely high-altitude areas of more than 3000 m, *O. sinensis* is mainly distributed in Qinghai Province and Tibet Province [4].

With increasing altitude, the most significant changes in ecological factors are the decrease in temperature, decrease in oxygen concentration, high radiation intensity, the high-temperature difference between day and night, and uneven precipitation between regions and months [5]. It has been reported that the temperature decreases by 0.53–0.78 °C for every 100 m increase in altitude [6], and the oxygen content decreases by 1.64% for every 1000 m increase [7]. Solar radiation also gradually changes from ultraviolet radiation (UV)-C (wavelength 200–280 nm) to mixed UV-B radiation (wavelength 280–320 nm) and UV-A (wavelength 320–400 nm) radiation [8]. This indicates that the growth environment of *O. sinensis* varies greatly at different altitudes. The difference in ecological factors caused by different growth environments is an important driving force for changes in organism species and shows adaptive evolution with the surrounding environment [9]. *Psammodromus algiruses* at high altitudes tend to have darker back colors, which they consider to be one of the survival strategies against low temperatures and UV radiation [10]. Rhododendrons adapt to low temperatures and water shortages by regulating photosynthesis, the osmotic pressure of leaf cells, and the synthesis of various oxidative protective agents [11]. *Pedicularis densispica* has adapted to environmental changes in high-altitude areas by reducing leaf length and width and increasing leaf thickness, mesophyll tissue thickness, and stomatal density [12].

*O. sinensis* has also undergone similar adaptive evolution. *O. sinensis* in Xiaojin County, Sichuan Province, China, is short and light yellow, with a small proportion of fruiting stroma and sclerotia. The fruiting stroma was rough in texture and had a mushroom-like flavor. The fruiting stroma and sclerotia of *O. sinensis* in Maqu County, Gansu Province, were longer, the fishy smell was clear, and the color was dark yellow. Most *O. sinensis* in Qinghai Province and Tibet Province are larger, the proportion of fruiting stroma and sclerotia is larger, with a thick fishy smell, and the color is bright yellow [13]. A study on the physiological and biochemical characteristics of *O. sinensis* in different producing areas showed that the color difference was related to the differential enrichment of anthocyanins and carotenoids in the fruiting stroma and sclerotia. The fruiting stroma accumulates a large amount of melanin due to exposure to air, making it black while chelating heavy metals and showing strong UV-blocking ability and antioxidant capacity [14,15]. The upregulated expression of cytochrome P450 *CYP61* in sclerotia promotes the synthesis of flavonoids and carotenoids, resulting in yellow sclerotia [16]. In recent years, it has been reported that different contents and activities of polysaccharides, total phenols, and flavonoids in different *O. sinensis* have led to differences in their antioxidant capacity [17,18,19]. Ergosterol, amino acids, and nucleosides vary with the growth cycle of *O. sinensis*, which is related to the complex metabolic regulation of *O. sinensis* in response to environmental stress [20]. In addition, mannitol content has been reported to be 10 times different in the two producing areas in Yushu City and Xiaojin County [21]. To date, there is still a lack of flavor omics research on *O. sinensis* from different habitats and growth environments. Volatile analysis based on headspace extraction technology may be an important means of understanding the unique odor composition of *O. sinensis* [22]. These results indicated that the appearance and physiological and biochemical characteristics of *O. sinensis* are affected by environmental factors. Unfortunately, the role of the environment and key environmental factors in the formation of *O. sinensis* has not been elucidated.

The factory-cultivated *O. sinensis* is always in a relatively stable laboratory-like environment, while the natural *Cordyceps sinensis* grows and develops in a high-altitude environment (>3000 m) and is always faced with a variety of environmental factors. Stress, such as low temperature, low oxygen, high solar radiation, day and night temperature conditions, etc. However, the effects and trends of environmental factors, such as soil factors and meteorological factors, on its nutrients have not been disclosed. The lack of these basic studies has led scholars to fail to understand the causes of the heterogeneity of natural and artificial *O. sinensis*. 

To study the effects of environmental factors on the metabolites of *O. sinensis* and to screen the relationship between key environmental factors and important metabolites. China is rich in *O. sinensis* resources. Among them, the *O. sinensis* in Xiaojin County, Sichuan Province, has been artificially domesticated and is suitable for large-scale artificial production with stable metabolite composition. It is an excellent material for studying the effects of environmental factors on *O. sinensis*, so we use it as a control group [23]. In addition, four groups of *O. sinensis* in the natural environment were set up in Yunnan Province, Gansu Province, Qinghai Province, and Tibet Autonomous Prefecture. Metabolomics and RDA were integrated to investigate the metabolite composition of *O. sinensis* from different production areas and their relationship with environmental factors, which provided a new perspective for understanding the metabolite composition and adaptive evolution of *O. sinensis* in different environments.

## 2. Materials and Methods

### 2.1. Sample Collection

**Figure 1:**
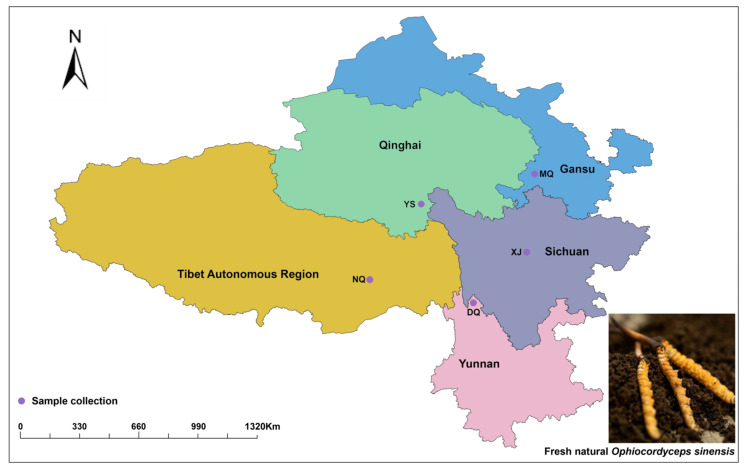
Geographical distribution map of five fresh natural *Ophiocordyceps sinensis*.

In 2023, from 5 June to 15 July 2023, the author commissioned Baohuitang Biological Company, Xining City, Qinghai Province, China, from Naqu City, Tibet Autonomous Region (NQ, 93°39′39″ N, 31.49582°29′45″ E, altitudes: 4390 m), Yushu Tibetan Autonomous Prefecture, Qinghai Province (YS, 97°7′56″ N, 33°22′59″ E, altitudes: 3885 m), Maqu County, Gannan Tibetan Autonomous Prefecture, Gansu Province (MQ, 102°11′47″ N, 34°3′16″ E, altitudes: 3583 m), Diqing Tibetan Autonomous Prefecture, Yunnan Province (DQ, 99°42′43″ N, 28°33′55″ E, altitudes: 3159 m) and Xiaojin County, Aba Tibetan and Qiang Autonomous Prefecture, Sichuan Province (XJ, 102°23′32″ N, 30°59′2″ E, altitudes: 2876 m) purchased *O. sinensis* samples with the same growth period, stored in liquid nitrogen and transported to the laboratory (Figure 1). At the same time, soil samples of 0–20 cm were taken from each producing area with a soil drill, sieved through a 60-mesh sieve, collected in a centrifuge tube, and stored in liquid nitrogen [24]. Six biological replicates were set in each region (sample of *O. sinensis* and soil). The *O. sinensis* samples from the five producing areas were mixed in equal amounts to make quality control (QC) samples, and the same treatment was performed with these five groups of samples to evaluate the stability of the instrument and balance the UPLC-MS/MS system.

### 2.2. Soil Factor Determination and Meteorological Factor Data Download

Environmental factors are generally divided into soil factors and meteorological factors. The related indexes of soil factors were determined as follows [25]. Organic matter (g/kg) was determined by the potassium dichromate–external heating method; total nitrogen (g/kg) was determined by the Kjeldahl method; total phosphorus (g/kg) was determined by the alkali fusion method; rapidly available phosphorus (mg/kg) was determined by the sodium bicarbonate extraction–colorimetric method; soil total potassium (g/kg) was determined by the acid-soluble method; rapidly available potassium (mg/kg) was determined by 1 mol/L ammonium acetate-flame determination of photometer; and rapidly available nitrogen (mg/kg) was determined by the alkaline hydrolysis diffusion method [24,26]. Fe (g/kg) was determined by phenanthroline colorimetry; Cu (mg/kg) was determined by flame atomic absorption spectrometry; Ca (g/kg) was determined by KMnO_4_ titration; Mg (g/kg) was determined by chelating titration; Na (g/kg), Mn (g/kg), and Zn (mg/kg) were determined by spectrophotometry; and the pH value was determined with an electronic pH meter [27,28]. Meteorological factors are obtained by the following methods. The laboratory logged into the China Climate Science Data Center (http://www.ncc-cma.net/) on 22 March 2024 and downloaded relevant meteorological data for these 5 regions in 2023. Detailed information and related abbreviations can be found in Table S1.

### 2.3. Sample Pretreatment and Metabolite Separation

Intact *O. sinensis* individuals (stroma and sclerotia) were used for the treatment and determination. The samples were washed three times with distilled water, and a filter paper was used to absorb the indicated water. Next, 100 mg was weighed and crushed with 6 mm diameter grinding beads and then added to 2 mL centrifuge tubes. The ground sample (50 mg) was added to 400 uL extractant (40% methanol + 40% acetonitrile + 20% water), mixed, and ground using a frozen tissue mill (JXFSTPRP-CLN-24, Shanghai, China). The parameters were set to –10 °C, 50 Hz, and 6 min. An ultrasonic cell pulverizer (SN-P650, Guangdong, China) was used for the extraction, and the parameters were set to 40 KHz, 5 °C, and 30 min. After that, the cytocentrifuge (JC-12L, Qingdao, China) was used for separation, and the parameters were set to 4 °C, 13,000 *g*, and 15 min. The supernatant was collected with a syringe and injected into the injection bottle through a 0.22 um filter membrane for detection.

### 2.4. UPLC-MS/MS Analysis

The methods for the extraction and detection of metabolites were modified based on existing research results in the laboratory [29,30]. The samples were analyzed by UHPLC-Q Exactive system with HSS T3 chromatographic column (100 mm × 2.1 mm i.d., 1.8 μm, Waters, Milford, CT, USA) and Thermo Q Exactive Focus mass spectrometer (Thermo Fisher Scientific, Waltham, MA, USA). The column temperature, injection volume, and flow rate were set at 30 °C, 3 uL, and 1 mL/min, respectively. Mobile phase A consisted of 95% water and 5% acetonitrile (containing 0.1% formic acid), and mobile phase B consisted of 47.5% acetonitrile, 47.5% isopropanol, and 5% water (containing 0.1% formic acid). The samples were analyzed using negative (NEG) and positive (POS) linear gradient elution. The linear gradient elution details were as follows: 0–0.5 min, 5% A/B; 0.5–6 min, 5–95% A/B; 6–7 min, 95–40% A/B; and 7–8 min, 5% A/B. The mass spectrometer parameters were set as follows: spray voltage ± 3500 V, sheath gas pressure 40 psi, auxiliary heating gas 10 psi, ion source heating temperature 400 °C, capillary temp (325 °C, orbitrap analyzer scan in the range of 70–1050 *m*/*z*, MS1 resolution 70,000 (full MS), and MS2 resolution 17,500.

### 2.5. Metabolite Identification and Data Analysis

The final result merges data in the NEG and POS modes. Proteo Wizard MSConvert converted the original data into MzXML format and then imported it into the XCMS program for baseline filtering, peak identification, integration, peak alignment, and retention time correction. The XCMS parameters are as follows: bw = 2, ppm = 15, peak width = c (5, 30), mzwid = 0.015, mzdiff = 0.01, and method = cent Wave. By discarding characteristic peaks with a relative standard deviation (RSD) > 30% in QC samples, a data matrix of retention time, mass-to-charge ratio, and peak intensity was obtained. The public databases HMDB, Massbank, McCloud, and KEGG and the self-built material library were used to identify the substances. The parameters were set to ppm 50% metabolites, extreme values were deleted, and the null values KNN (the minimum value in the original matrix) were filled. To reduce the error caused by the sample preparation process and the instrument, the response intensity of the sample mass spectrum peak was normalized by the total peak area and Log_10_ logarithm, and the data matrix for subsequent analysis was obtained.

Principal component analysis (PCA) and orthogonal partial least squares discriminant analysis (OPLS-DA) were performed on the sample data using the R software package Ropls (Version1.6.2). The model was tested using 200 permutations. R^2^X and R^2^Y represent the interpretation rates of the model for X and Y matrices, respectively. Q^2^ indicated the predictive ability of the model. The closer their values are to 1, the better the fitting degree of the model. Based on the OPLS-DA dimension reduction method, the variable importance in projection (VIP) and fold change (FC) were calculated to calculate the difference multiples between groups, and the influence of intensity and interpretation ability of each metabolite component content on sample classification discrimination were measured to assist in the screening of metabolites. When the *p*-value < 0.5, the metabolite molecules are considered to be statistically significant. The Metabo Analyst software package (metaboanalyst.ca, 6.0) was used to perform functional pathway enrichment and topology analyses of the screened differential metabolic molecules. The enriched pathways were browsed using the KEGG Mapper visualization tool for differential metabolites and pathway maps [31].

## 3. Results

### 3.1. Environmental Factor Analysis

**Figure 2:**
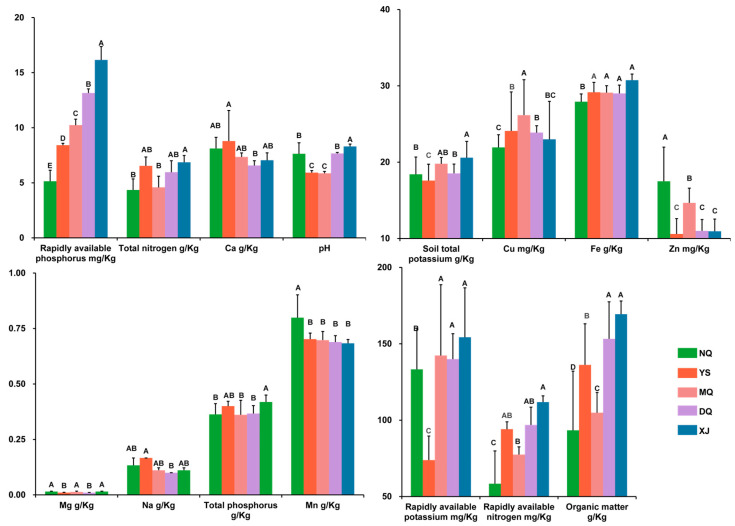
Soil condition analysis of different production areas. The line segment represents the positive standard error, and the difference level of different capital letters is *p* < 0.05.

The altitudes of XJ, DQ, MQ, YS, and NQ gradually increased, whereas the mean annual precipitation, wet season precipitation, wettest monthly precipitation, and warmest season mean temperature gradually decreased. The variation ranges of the annual average temperature and monthly mean diurnal temperature difference gradually increased (Table S1). In terms of soil factors, XJ had the highest rapidly available phosphorus, rapidly available potassium, rapidly available nitrogen, total nitrogen, total potassium, total phosphorus, and pH (*p* < 0.05), whereas NQ had the lowest. Ca, Cu, Fe, Zn, Mg, Na, and Mn showed different significant levels among the samples (Figure 2). In general, there were significant differences in meteorological and soil conditions among the five *O. sinensis*-producing areas. The environmental conditions of XJ were relatively good, whereas those of DQ and YS were poor.

### 3.2. Total Metabolic Spectrum Structure of O. sinensis

**Figure 3:**
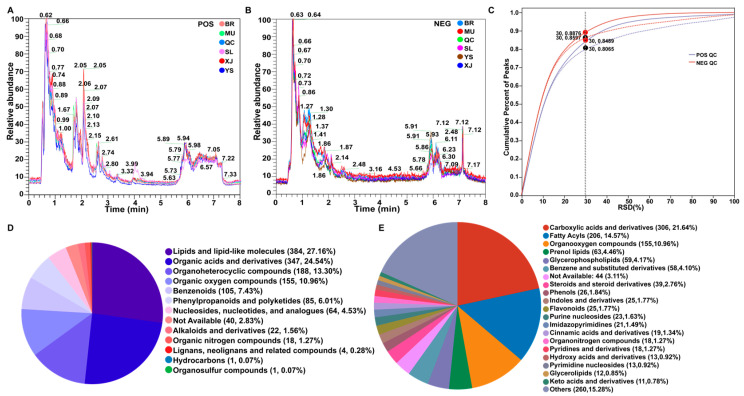
Analysis of total metabolite characteristics. Total ion chromatograms of all samples ((**A**) POS; (**B**) NEG). (**C**) RSD test diagram of the samples. Classification pie charts for all metabolites ((**D**) Superclass; (**E**) Class).

The composition of the metabolites of *O. sinensis* was determined to evaluate changes in the structure of *O. sinensis* metabolites in different producing areas. QC samples were obtained by combining the five groups of samples to investigate the stability and accuracy of the UPLC-MS/MS system. In the POS and NEG modes, the retention time, peak shape, and peak intensity of the whole chromatogram had good repeatability (Figure 3A,B), and the relative standard deviation reached 0.80 (Figure 3C) when <30%, indicating that the data have excellent stability and reliability. A total of 2223 ion peaks (POS: 1113, DEG: 1110) (Table S2) were detected in the five groups of *O. sinensis* samples, and 1414 compounds (POS: 662, NEG: 752) (Table S3) were obtained after annotation. They were divided into 13 superclasses, of which the top five were lipids and lipid-like molecules (*n* = 384, 27.16%), organic acids and derivatives (*n* = 347, 24.54%), and organoheterocyclic compounds (*n* = 188, 13.30%). Organic oxygen compounds (*n* = 155, 10.96%) and benzenoids (*n* = 105, 7.43%) (Figure 3D). They were also divided into 118 categories, of which the top five were carboxylic acids and derivatives (*n* = 306, 21.64%), fatty acyls (*n* = 206, 14.57%), organooxygen compounds (*n* = 155, 10.96%), and prenol lipids (*n* = 63, 4.46%). Glyerophospholipids (*n* = 59, 4.17%), benzene, and substituted derivatives (*n* = 58, 4.10%) (Figure 3E).

### 3.3. Multivariate Statistical Analysis and DAMs Identification

**Figure 4:**
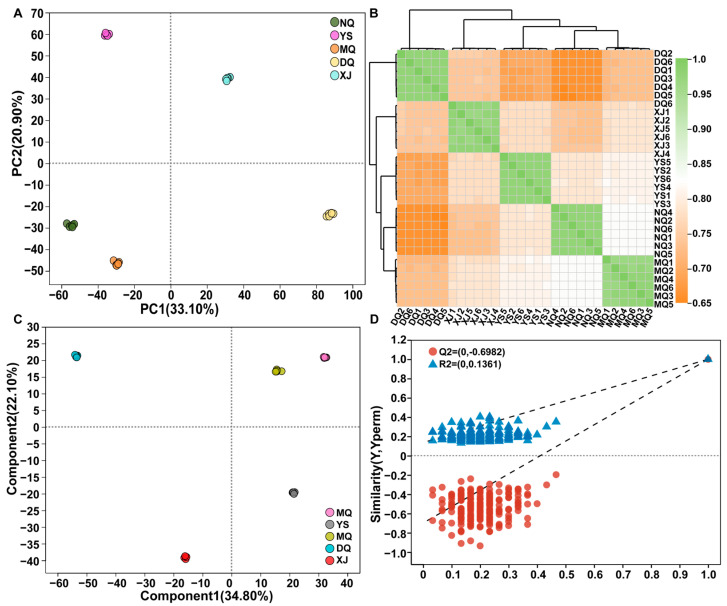
Multivariate statistical analysis of *Ophiocordyceps sinensis*. (**A**) PCA score plot of all samples. (**B**) Correlation analysis of all samples. (**C**) OPLS-DA score plots for all the samples. (**D**) The OPLS-DA model was based on a permutation test graph of 200 iterations.

Multivariate statistical analysis was used to determine the structure of *O. sinensis* metabolites from different habitats. PCA (PC1 = 33.10%, PC2 = 20.90%) divided the samples into five groups (Figure 4A) with a long distance between groups and a good biological repeat aggregation effect in the group. Pearson’s correlation analysis (Figure 4B) revealed that the metabolites of the five groups of *O. sinensis* were quite different, and the similarity of metabolites in the group was high. The OPLS-DA model was used to test the accuracy of PCA. The results showed that the OPLS-DA model score was 56.90% (Figure 4C). Based on 200 model validation replicates, R^2^ = 0.698 and Q^2^ = 0.136 (Figure 4D). At the same time, pairwise comparisons between all *O. sinensis* samples: NQ vs. XJ (Figure S1A,B), MQ vs. XJ (Figure S1C,D), DQ vs. XJ (Figure S1E,F), YS vs. XJ (Figure S1G,H), show that the Q^2^ value is always less than the R^2^ value, indicating that the model fits well and does not over-fit, with good interpretability and predictability.

**Figure 5:**
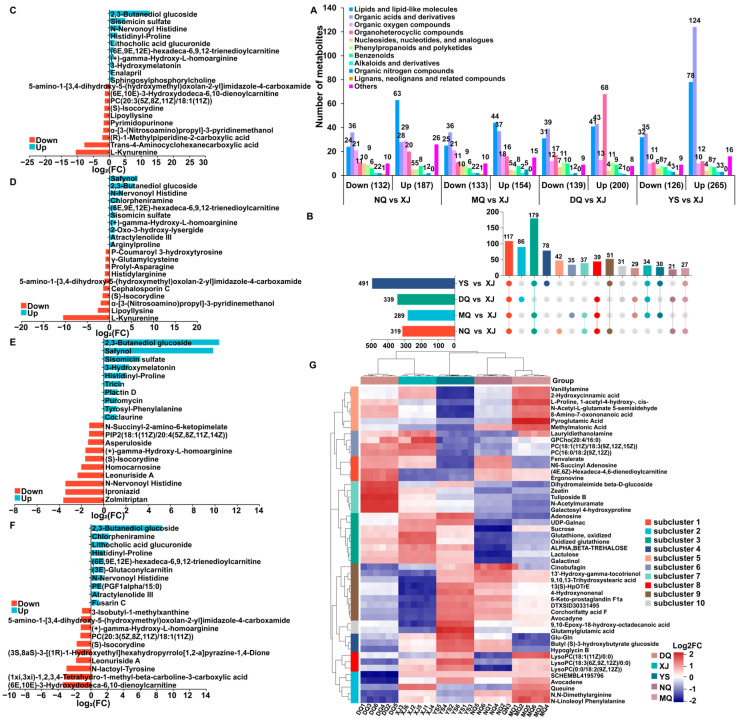
Identification and analysis of DAMs. (**A**) Distribution histograms of all DAMs between the samples. (**B**) Venn diagram of DAMs in the different comparison groups. VIP random forest maps of the top 20 DAMs between the different comparison groups ((**C**) NQ vs. XJ, (**D**) MQ vs. XJ, (**E**) DQ vs. XJ, (**F**) YS vs. XJ). (**G**) HCA diagram of VIPTop50DAMs among different samples. SCHEMBL4195796: (2R, 4S)-1-(tert-butoxycarbonyl)-4-phenylpyrrolidine-2-carboxylic acid; dTXSID30331495: 3-Oxo-2-(2-entenyl) cyclopentaneoctanoic acid.

To further understand the variation characteristics of *O. sinensis* metabolites in different production areas, the VIP value was calculated based on the scores of each metabolite in the OPLS-DA model. DAMs were screened with VIP ≥ 1.5 and *p* < 0.5, and a total of 700 DAMs (POS: 330, NEG: 370) were screened. In NQ vs. XJ, 319 DAMs (187 upregulated and 132 downregulated) were identified, of which most lipids and lipid-like molecules (72.41%) and organoheterocyclic compounds (64.52%) were upregulated, such as histidinyl-proline, 3-Hydroxymelatonin, and lithocholic acid glucuronide. At the same time, most of the organic acids and derivatives (56.25%), nucleosides, nucleotides, and analogs (66.67%), and phenylpropanoids and polyketides (64.29%) were downregulated, such as 5-amino-1-[3,4-dihydroxy-5-(hydroxymethyl)oxolan-2-yl]imidazole-4-carboxamide, (S)-isocorydine, and lipoyllysine (Figure 5C). In MQ vs. XJ, 289 DAMs (154 upregulated and 133 downregulated) were identified, most of which were lipids and lipid-like molecules (63.77%) and organoheterocyclic compounds (59.26%), such as arginylproline. N-Nnervonoyl histidine and 2-oxo-3-hydroxy-lysergide. Most nucleosides, nucleotides, and analogs (53.33%) and phenylpropanoids and polyketides (69.23%) were downregulated, including 5-amino-1-[3,4-dihydroxy-5-(hydroxymethyl)oxolan-2-yl]imidazole-4-carboxamide, (S)-isocorydine, lipoyllysine, and L-kynurenine (Figure 5D). In DQ vs. XJ, a total of 339 DAMs (200 upregulated and 139 downregulated) were identified, most of which were lipids and lipid-like molecules (56.94%) and organoheterocyclic compounds (80.00%) were upregulated, such as histidinyl-proline and tyrosyl-phenylalanine. Puromycin, and 3-hydroxymelatonin (Figure 3E). A total of 491 DAMs (265 upregulated and 126 downregulated) were identified in YS vs. XJ, of which most lipids and lipid-like molecules (70.91%) and organic acids and derivatives (77.99%), such as histidinyl-proline, were upregulated. Lithocholic acid glucuronide, and N-nervonoyl histidine (Figure 5A,F).

A total of 117 DAMs were shared among the four comparison groups, and 42, 35, 86, and 78 unique DAMs were found in NQ vs. XJ, MQ vs. XJ, DQ vs. XJ, and YS vs. XJ, respectively (Figure 5B). These results indicate that DAMs in *O. sinensis* from different habitats had different accumulation patterns, and they were mainly lipids and lipid-like molecules (21.24–27.27%), organic acids and derivatives (20.06–32.38%), organoheterocyclic compounds (4.68–25.07%), and nucleosides, nucleotides, and analogs (2.04–5.19%). YS had the most abundant lipids and lipid-like molecules, organic acids, and derivatives; the diversity of organic oxygen compounds in NQ was the best. DQ contains the most organoheterocyclic compounds, and MQ contains the most nucleosides, nucleotides, and analogs compounds. Notably, lipids and lipid-like molecules were upregulated in the four comparisons, which may be an important reason for the differences in the metabolites of *O. sinensis* in different producing areas.

HCA was performed on the top 50 important DAMs of the VIP value to determine the accumulation pattern of DAMs from different producing areas. In DQ, the metabolites of subcluster 2 and subcluster 8 showed a downward trend, and the metabolites of subcluster 7 and subcluster 1 showed an upward trend. In MQ, the metabolites of subcluster 4 and subcluster 7 showed a downward trend, and the metabolites of subcluster 5 and subcluster 2 showed an upward trend. In NQ, the metabolites of subcluster 8, subcluster 3, and subcluster 6 showed a downward trend, and the metabolites of subcluster 9 and subcluster 1 showed an upward trend. In YS, the metabolites of subcluster 5, subcluster 6, and subcluster 8 showed a downward trend, and the metabolites of subcluster 4 and subcluster 10 showed an upward trend. In XJ, the metabolites of subcluster 9 and subcluster 10 showed a downward trend, and the metabolites of subcluster 3 and subcluster 6 showed an upward trend (Figure 5G). This means that the composition and content of metabolites of *O. sinensis* in different production areas are quite different, and they are mainly lipids, lipid-like molecules, organic acids, and derivatives.

### 3.4. KEGG Enrichment Analysis

**Figure 6:**
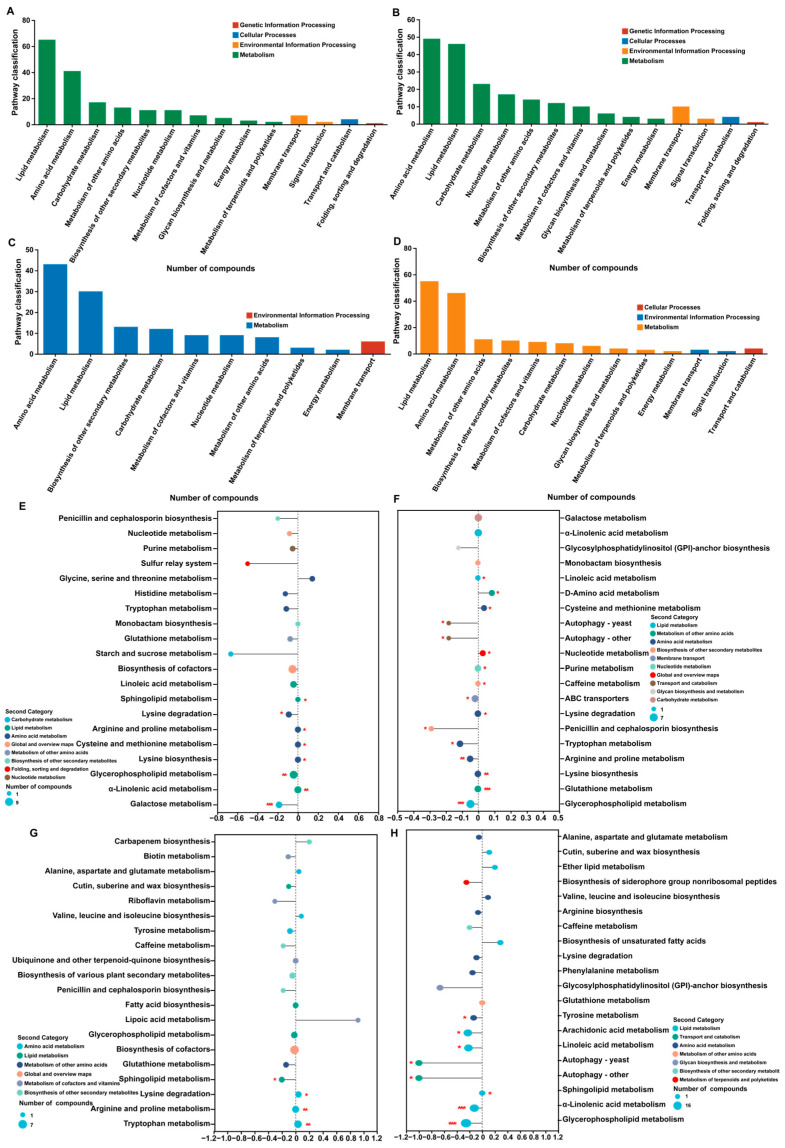
KEGG enrichment maps of the DAMs. KEGG functional enrichment maps of the DAMs ((**A**) NQ vs. XJ, (**B**) MQ vs. XJ, (**C**) DQ vs. XJ, (**D**) YS vs. XJ). KEGG enrichment pathways DA score map of DAMs ((**E**) NQ vs. XJ, (**F**) MQ vs. XJ, (**G**) DQ vs. XJ, (**H**) YS vs. XJ). DA score reflects the overall changes in all metabolites in the metabolic pathway. DA score > 0 indicates that the expression trend of all annotated differential metabolites in the pathway is upregulated, DA score < 0 indicates that the expression trend of all annotated differential metabolites in the pathway is downregulated, and the length of the line segment indicates the absolute value of DA score.

In the enrichment analysis of KEGG functional pathways in the four composition pairs, significantly enriched metabolic pathways were selected at *p* < 0.05. These metabolic pathways have four distinct functions, namely, genetic information processing, cellular processes, environmental information processing, and metabolism. The four component pairs shared amino acid metabolism, biosynthesis of other secondary metabolites, carbohydrate metabolism, energy metabolism, lipid metabolism, and nucleotide metabolism. Only membrane transport was shared during environmental information processing (Figure 6A–D). This indicates that membrane transport is the main pathway for *O. sinensis* to respond to environmental changes and that it changes the metabolic processes of amino acids, carbohydrates, lipids, and nucleotides to adapt to environmental changes. In the KEGG pathway enrichment analysis, the four composition pairs shared tryptophan metabolism, lysine degradation, glycerophospholipid metabolism, and glutathione metabolism, among which DAMs involved in lysine degradation were significantly downregulated (*p* < 0.05) (Figure 6E–H). This indicates that the metabolic intensity and accumulation trend of DAMs in lysine degradation are important reasons for the differences in the metabolites of *O. sinensis* in different production areas. In conclusion, membrane transport and lysine degradation are key pathways for *O. sinensis* to cope with environmental changes by affecting the content and composition of amino acids, carbohydrates, lipids, and nucleotides.

### 3.5. Association Analysis between Environmental Factors and DAMs

**Figure 7:**
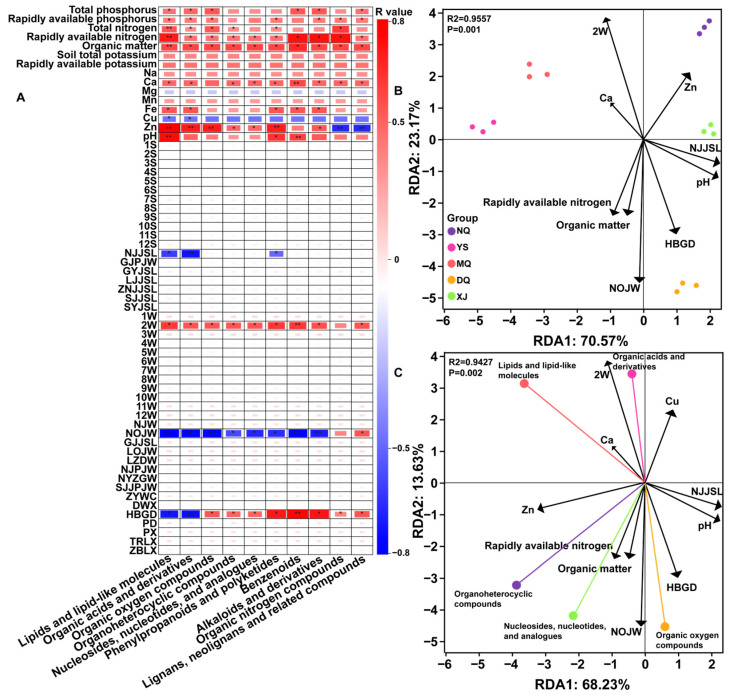
Analysis of reverse correlation between environmental factors and metabolites. (**A**) Pearson correlation analysis between environmental factors and the number of DAMs. ‘*’ means the significance level was *p* < 0.05; ‘**’ means the significance level is *p* < 0.01. (**B**) RDA analysis of important environmental factors and the number of DAMs in all superclass metabolites of *O. sinensis*. (**C**) RDA analysis of important environmental factors and the number of DAMs of the first five superclass metabolites. Black arrows represent environmental factors, and colored arrows represent different superclass metabolites. Abbreviations of these environmental factors can be found in Table S1.

By constructing a correlation matrix between environmental factors and the number of metabolites, the environmental factors significantly related to DAMs were found. The results showed that 12 important environmental factors, such as 2W, 3W, 3S, NOJW, HBGD, pH, and organic matter, were significantly positively or negatively correlated with most metabolites (*p* < 0.05) (Figure 7A). Nine important environmental factors cumulatively explained 93.74% (RDA1 = 70.57%, RDA2 = 23.17%, R = 0.9557, *p* < 0.05) of the variation characteristics of *O. sinensis* metabolites in different producing areas. 2W, 3W, and NJJSJ were the main positive factors affecting the characteristics of NQ *O. sinensis* metabolites (*p* < 0.05). HBGD, NOJW, and organic matter were the main negative factors that inhibited the accumulation of NQ *O. sinensis* metabolites (*p* < 0.05). Rapidly available nitrogen, organic matter, 2W, and 3W were the main positive factors affecting the characteristics of YS *O. sinensis* metabolites (*p* < 0.05). NJJSL, pH, and HBGD were the main negative factors that inhibited the accumulation of YS metabolites (*p* < 0.05). 3W, 2W, and 3S were the main positive factors affecting the characteristics of MQ *O. sinensis* metabolites (*p* < 0.05). NJJSL, pH, NOJW, and HBGD were the main negative factors that inhibited the accumulation of MQ *O. sinensis* metabolites (*p* < 0.05). HBGD, pH, NJJSL, and NOJW were the main positive factors affecting the characteristics of DQ *O. sinensis* metabolites (*p* < 0.05). 2W, 3W, and 3S were the main negative factors that inhibited the accumulation of DQ *O. sinensis* metabolites (*p* < 0.05). NJJSL, pH, and 3S were the main positive factors affecting the metabolite characteristics of XJ *O. sinensis* (*p* < 0.05). NOJW, rapidly available nitrogen, and organic matter were the main negative factors that inhibited the accumulation of metabolites in XJ (*p* < 0.05) (Figure 7B). These results indicate that these environmental factors are important factors leading to the characteristics of *O. sinensis* metabolites in different producing areas.

To further understand the effects of environmental factors on different superclass metabolites, the top five superclass metabolites and environmental factors were used for RDA in this study (RDA1 = 68.23%, RDA2 = 13.67%, R = 0.9427, *p* < 0.05) (Figure 7C). 2W, 3W, and NJW significantly promoted the accumulation of lipids and lipid-like molecules and organic acids and derivatives. NOJW, NJJSL, pH, and HBGD significantly inhibited the accumulation of lipids and lipid-like molecules and organic acids and derivatives (*p* < 0.05). Rapidly available nitrogen, organic matter, NOJW, and pH significantly promoted the accumulation of organoheterocyclic compounds and nucleosides, nucleotides, and analogs; 2W and 3S significantly inhibited the accumulation of organoheterocyclic compounds and nucleosides, nucleotides, and analogs (*p* < 0.05). Organic matter, rapidly available nitrogen, HBGD, pH, NOJW, and NJJSL significantly promoted the accumulation of organic oxygen compounds. 2W, 3W, and 3S significantly inhibited the accumulation of organic oxygen compounds (*p* < 0.05). These results indicate that the different effects of these environmental factors on the superclass metabolites are an important source of differences in the metabolic profiles of *O. sinensis* in different producing areas.

## 4. Discussion

Metabolites are not only participants in the physiological and biochemical processes and morphological composition of organisms but also play an important role in the process of individual resistance to abiotic stress [32]. Great differences in ecological factors have led to dramatic changes in the metabolic landscape of *O. sinensis* in different regions. *O. sinensis* formed under such extreme environmental conditions is considered to be a genuine medicinal material with more extraordinary secondary metabolites, higher nutritional quality, and medicinal value. It is an ideal tonic for the maintenance of life function in Chinese medicinal materials and the health care of sub-healthy people [33]. In this study, we found that multiple environmental factors, such as 3W, 3S, NJJSL, pH, NOJW, and HBGD, played an important role in the composition of primary metabolites and the formation of secondary metabolites in *O. sinensis* (Figure 7B). Among them, lipids and lipid-like molecules, organic acids and derivatives, organoheterocyclic compounds and nucleosides, nucleotides, and analogs were more sensitive to environmental changes (Figure 7C).

Lipids and lipid-like molecules have been reported to have a variety of biologically active functions [34]. The immune function of mice fed with methanol extract of Cordyceps sinensis was activated and amplified, and the activities of SOD, catalase, and GSEH-px in liver cytoplasm increased significantly, showing strong antioxidant capacity [35]. A novel fatty acid, (2Z, 4E)-deca-2,4-dienoic acid (DDEA), attenuates pro-inflammatory responses by inhibiting mRNA transcription and protein synthesis in macrophages derived from A549 and U937 cells while downregulating *TLR-3*, *RIG-I*, and *IFN*-activated innate immune signaling pathways to exert anti-inflammatory effects [36]. Amino acids in organic acids and derivatives have been reported to effectively improve cyclophosphamide-induced bone marrow hematopoietic factor reduction, pathological mutations, and bone marrow apoptosis, which depends on their rich content of L-pyroglutamic acid, lysinonorleucine, tryptophan, and phenylalanine [37]. Glutamate and arginine are also considered to be important components for their antibacterial and anti-inflammatory properties [38]. This study found that YS has the most abundant lipids and lipid-like molecules and organic acids and derivatives, which indicates that the *O. sinensis* of YS has great potential for anti-inflammatory effects, enhancing immunity, and bone marrow protection. It is also one of the functional foods for consumers to regulate their autoimmune function. February average temperature (2W), march average temperature (3W), and the variation range of annual average temperature (NJW) promoted their accumulation and were inhibited by NOJW, NJJSL, pH, and HBGD. It has been reported that temperature stress promotes the upregulation of two environmental sensing factors, the pheromone receptor gene (*OSIN6252*) and amino acid sensing gene (*OSIN6398*), and the mitogen-activated protein kinase (MAPK) signaling pathway in *Cordyceps sinensis*, and inhibits the accumulation of amino acids in stroma and sclerotia [39]. In the extremely high-altitude area above 2000 m, with the increase in HBGD, NJW gradually increased, NOJW gradually decreased, and NJJSL gradually decreased (−10.7–15 °C) (Table S1). This is not suitable for the growth and development of *O. sinensis*. To resist the low-temperature environment, the fungal cell membrane has evolved an interwoven membrane lipid structure to maintain the fluidity of the cell membrane [40]. Secondly, high altitude creates a hypoxic natural environment, glycolysis and tricarboxylic acid cycle (TCA) cycle processes are inhibited, and *O. sinensis* initiates anaerobic respiration to obtain sufficient energy supply, resulting in lipids and-like lipid molecules. Compounds are decomposed into small molecular weight lipid secondary metabolites [41]. Anaerobic respiration leads to the production of singlet oxygen, superoxide, and lipid peroxide, which leads to the loss of cell membrane function or local damage. Lipids and lipid-like molecules have strong antioxidant capacity and free radical scavenging ability [42], which promotes *O. sinensis* to adapt to low-temperature environments. The period from February to April of each year is the period of germination and development of *O. sinensis* stroma, which is an important stage for the formation of its nutritional quality [21]. At this time, the temperature of the Qinghai–Tibet Plateau increased, and NJW became larger [43], which was conducive to the germination and development of *O. sinensis*, which also explained the positive correlation between 2W, 3W, and NJW and lipids and lipid-like molecules in this study.

Organoheterocyclic compounds and nucleosides, nucleotides, and analogs have been reported for the treatment of cancer and kidney diseases. It has been reported that double heterocyclic compounds can be used as drug carriers to target and identify cancer cells to exert anticancer effects [44,45]. Studies have shown that the nucleosides of *O. sinensis* reduce the deposition of EMT and ECM by inhibiting p38 and ERK signaling pathways and play a therapeutic role in diabetic renal fibrosis [46]. It has also been reported that nucleosides inhibit chronic obstructive pulmonary disease inflammation and maintain lung function by upregulating the SIRT1-NF-κB/p65 pathway [47]. This study found that DQ and MQ have the most abundant organoheterocyclic compounds and nucleosides, nucleotides, and analogs, respectively. This indicates that DQ and MQ *O. sinensis* are excellent raw materials and excellent medicinal materials for the treatment of cancer and kidney diseases. Rapidly available nitrogen, organic matter, NOJW, and pH were the promoting factors for organoheterocyclic compounds and nucleosides, nucleotides, and analogs accumulation. March precipitation (3S) and 2W are inhibitors of organoheterocyclic compounds and nucleosides, nucleotides, and analogs accumulation. The contents of rapidly available nitrogen and organic matter in YS and NQ soils were low, and the comprehensive soil conditions were poor (Figure 2), which could only support the growth of low herbaceous plants and shrubs. DQ and MQ have better soil conditions to promote the formation of tree community structure, provide a better humidity environment and heat preservation effect while also shielding solar radiation [48], and provide a suitable environment for the growth and development of *O. sinensis* to indirectly promote the accumulation of organoheterocyclic compounds and nucleosides, nucleotides, and analogs. These influencing factors are not fixed in the growth and development of *O. sinensis* but are constantly changing and have different effects at different stages [49]. The climate of the Qinghai–Tibet Plateau gradually warms up in February and March every year, and it is warming. The vegetation is dominated by herbaceous plants, the tall trees are still in the regreening period, and the precipitation is locally aggregated on the spatial scale [50,51]. At this time, 2W and 3S provided suitable temperature and humidity conditions for the stroma of *O. sinensis* to promote its germination, and polysaccharides, polyphenols, and mannitol were synthesized in large quantities, inhibiting the accumulation of organoheterocyclic compounds and nucleosides, nucleotides, and analogs [52].

In summary, environmental factors induce the differential accumulation of lipids and lipid-like molecules, organic acids and derivatives, organoheterocyclic compounds and nucleosides, nucleotides, and analog metabolites in *O. sinensis* to adapt to environmental changes. These environmental factors indirectly lead to differences in the nutritional quality and medicinal value of *O. sinensis* in different producing areas. In this study, nine environmental factors that had significant effects on the metabolites of *O. sinensis* were found, and their effects on the composition of *O. sinensis* metabolites were discussed, which provided important parameters for its artificial culture. Perhaps in the future, we may promote the biosynthesis of some active substances in *O. sinensis* by artificially controlling certain environmental factors so as to provide excellent raw materials for medical workers and pharmacists. It provides a more direct scientific basis for its edible and medicinal selection and also provides new insights for us to understand the response of *O. sinensis* to environmental metabolites.

## 5. Conclusions

Metabolomics and RDA were integrated to find the metabolite profile characteristics and key environmental factors of *O. sinensis* in different producing areas. The results showed that the accumulation patterns of *O. sinensis* metabolites in different producing areas were quite different, and 319 (NQ vs. XJ), 289 (MQ vs. XJ), 339 (DQ vs. XJ), and 491 (YS vs. XJ) DAMs were identified. HCA and KEGG analysis of these DAMs showed that lipids and lipid-like molecules, organic acids and derivatives, and organoheterocyclic compounds were involved in tryptophan metabolism and lysine degradation. The differential expression of glycerophospholipid metabolism and glutathione metabolism pathways is the main reason for the differential accumulation of *O. sinensis* metabolites in different producing areas. Pearson correlation and RDA between metabolites and environmental factors revealed that nine environmental factors, including NJJSL, pH, NOJW, and HBGD, interacted with lipids and lipid-like molecules, organic acids and derivatives, organoheterocyclic compounds and nucleosides, and nucleotides. The abundance of analogs had a significant influence (R = 0.9557, *p* < 0.05), which made *O. sinensis* in different growing environments in different producing areas show different metabolic spectrum characteristics. These results provide new insights into the stress response of *O. sinensis* to the environment and the evolution of metabolites.

## Data Availability

All data are included in this manuscript and the supplementary materials, which are freely available from the corresponding author.

## Supplementary Materials

The following supporting information can be downloaded at https://www.mdpi.com/article/10.3390/metabo14080414/s1, Figure S1: OPLS-DA analysis of different comparison groups (A, NQ vs. XJ; C, MQ vs. XJ; E, DQ vs. XJ; G, YS vs. XJ); 200 permutation test charts of different comparison groups (B, NQ vs. XJ; D, MQ vs. XJ; F, DQ vs. XJ; H, YS vs. XJ). Table S1: Detailed environmental factor information for *Ophiocordyceps sinensis* samples in 2023; Table S2: Details of all compounds detected in different *Ophiocordyceps sinensis* samples; Table S3: Compounds identified from different *Ophiocordyceps sinensis* samples and their taxonomy.
