# Supplementary figures and images for: Accumulation Characteristics of Natural Ophiocordyceps sinensis Metabolites Driven by Environmental Factors
Source: Metabolites. 2024 Jul 27;14(8):414. doi: 10.3390/metabo14080414 (PMC11355974; doi:10.3390/metabo14080414)

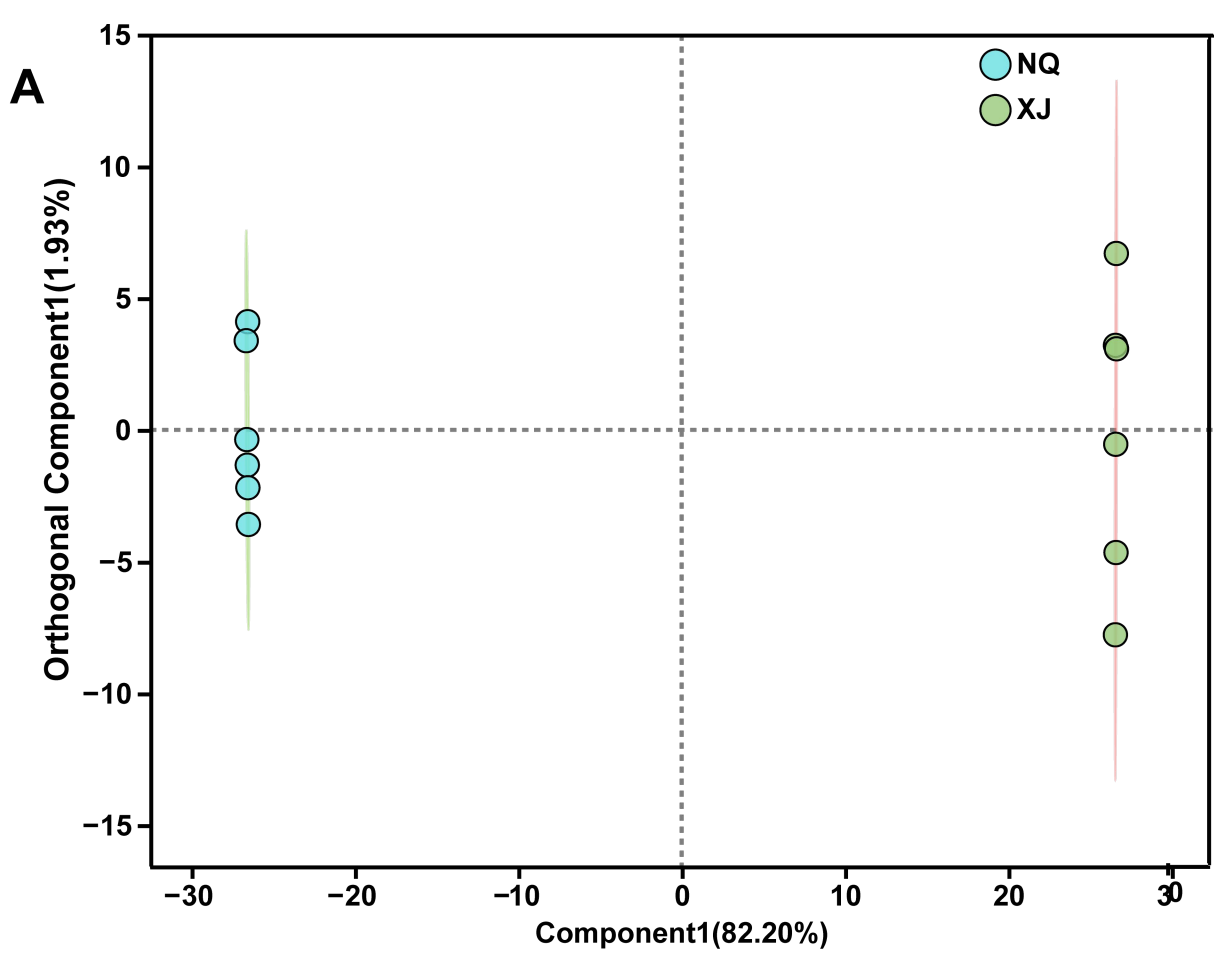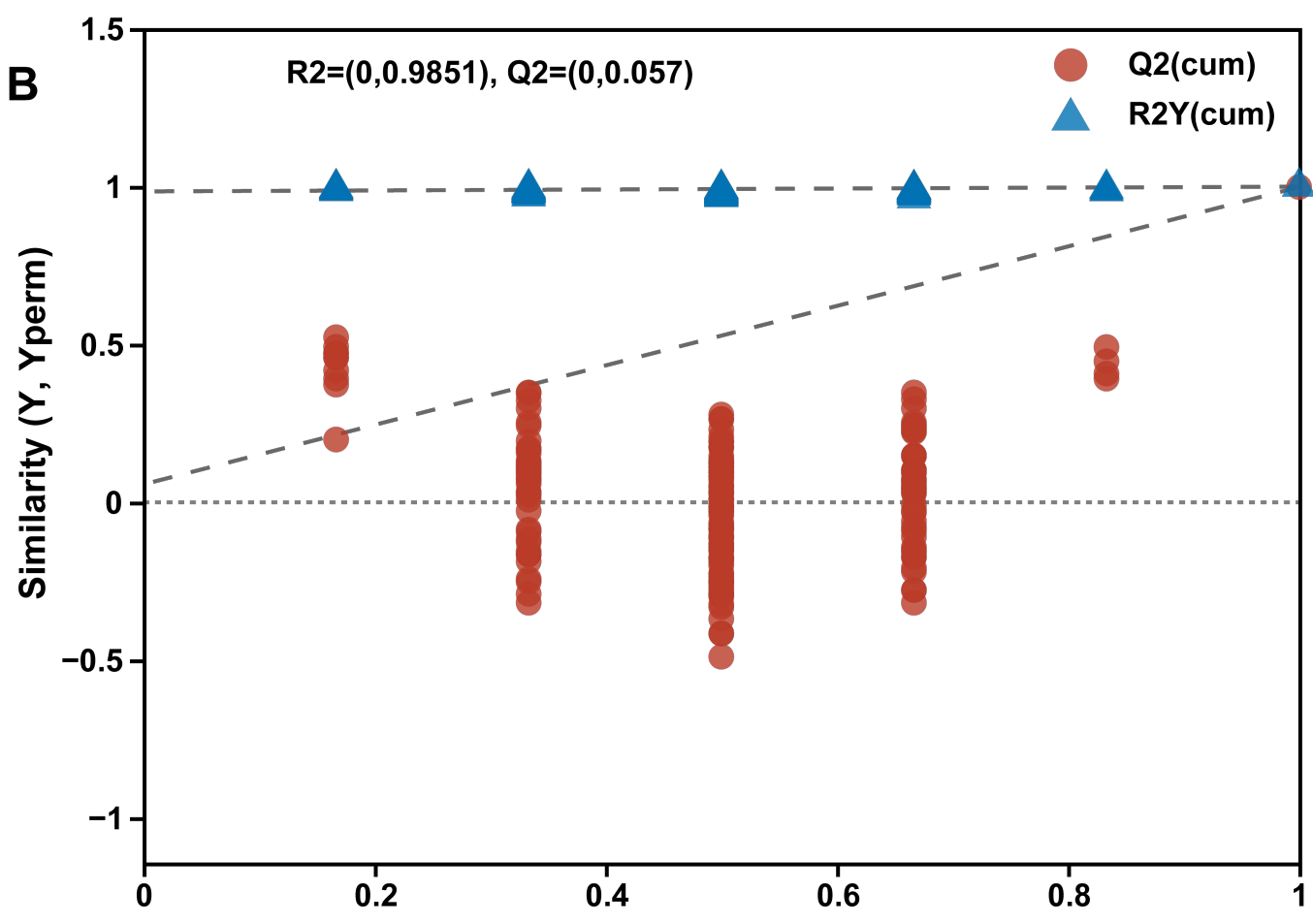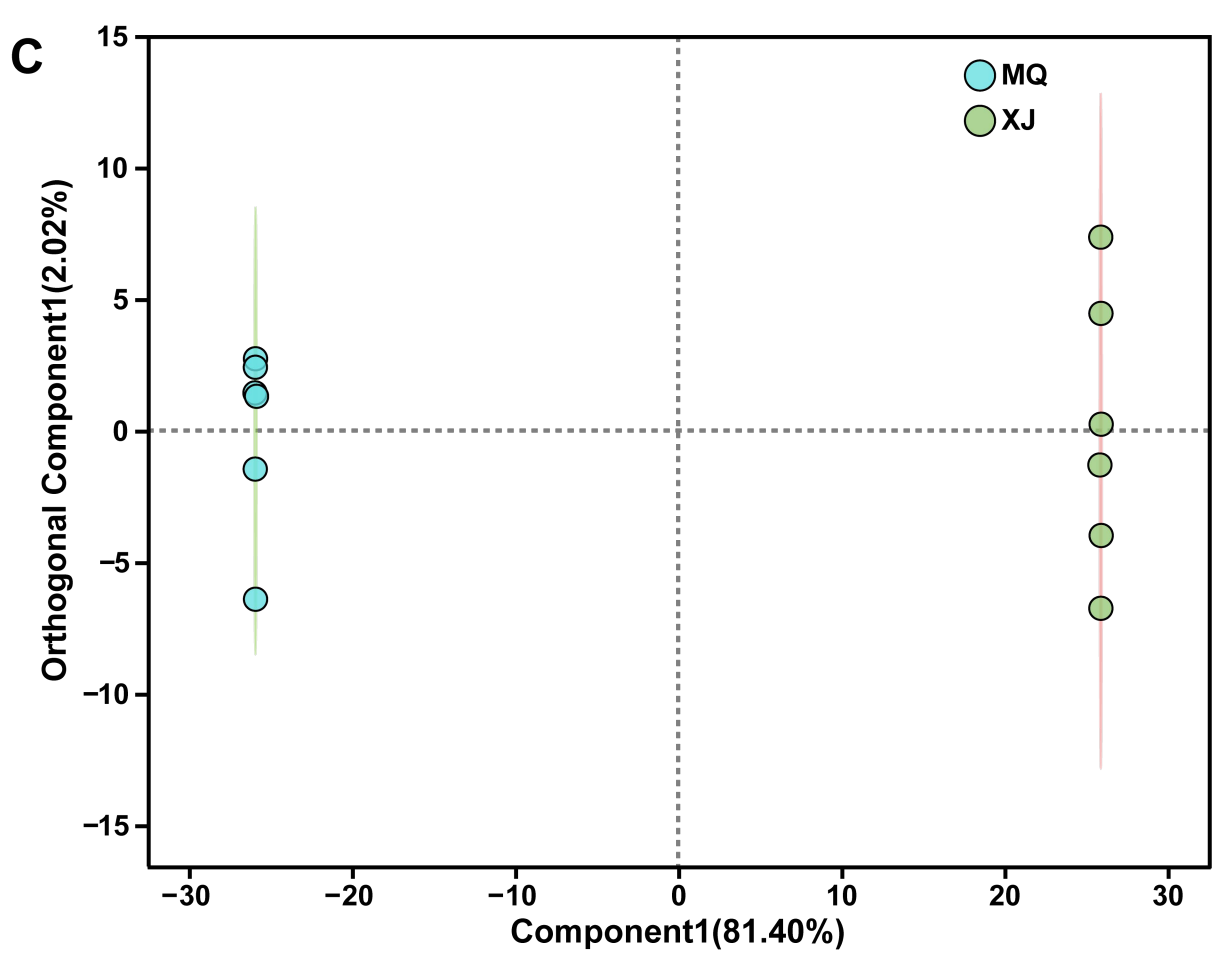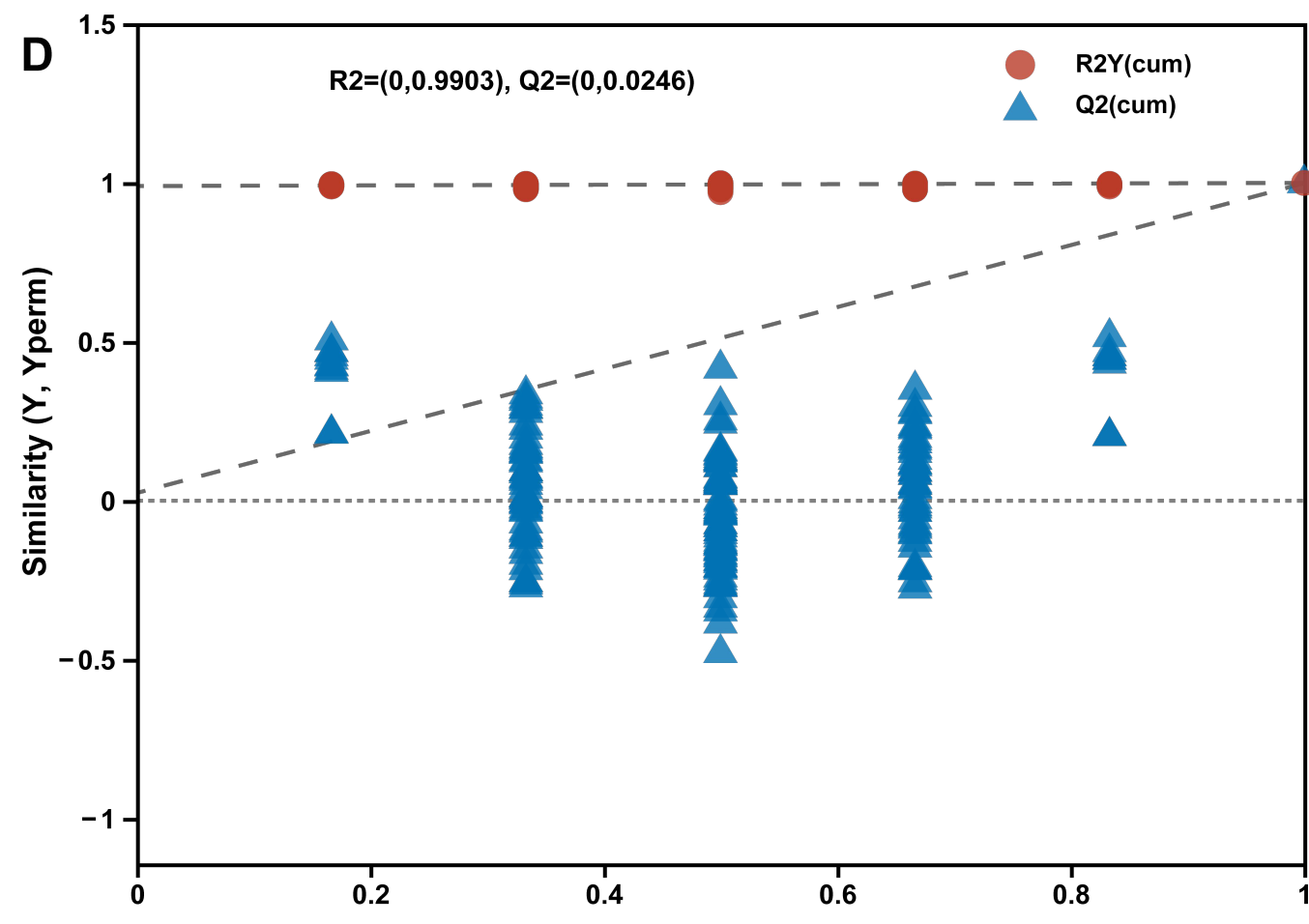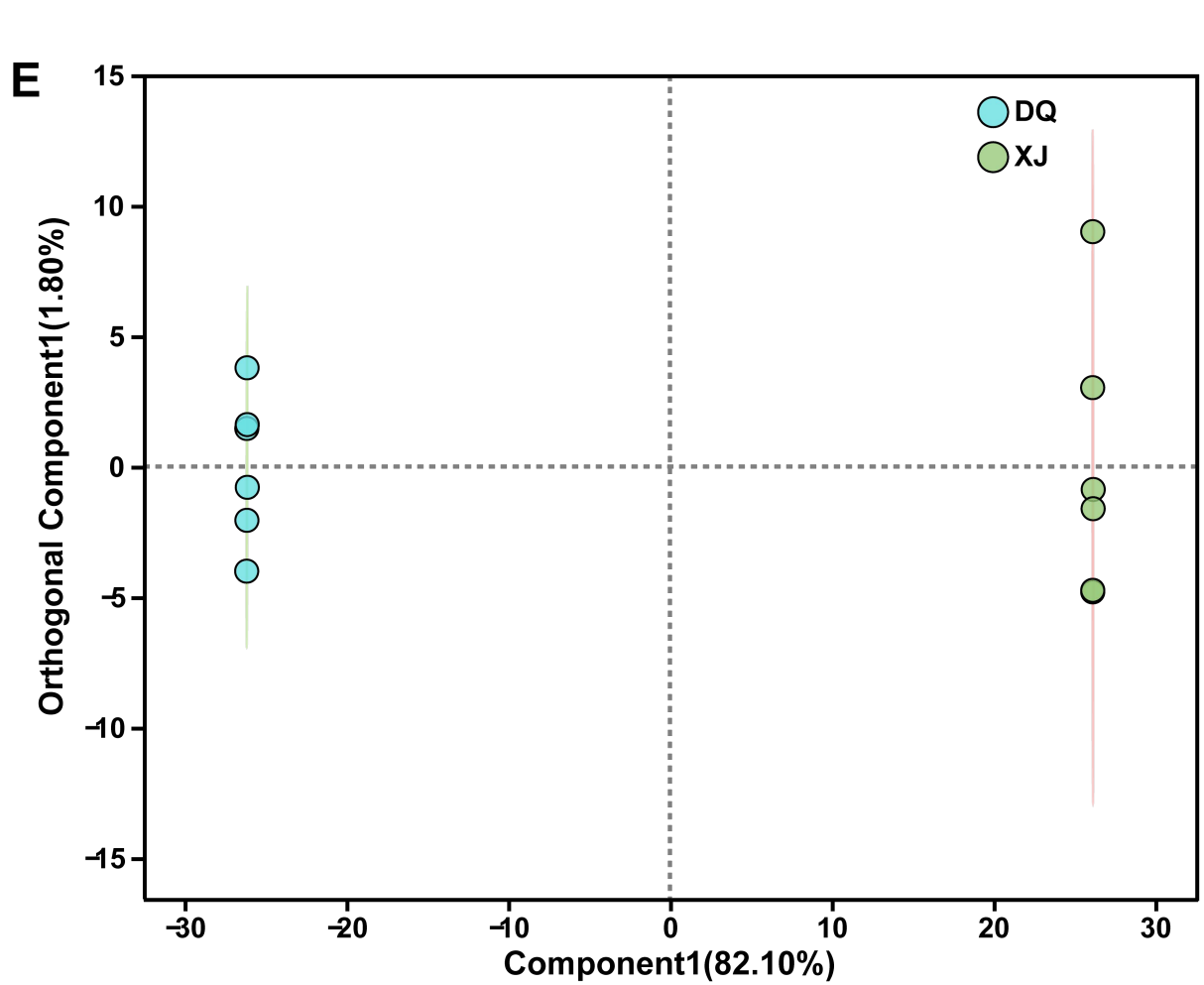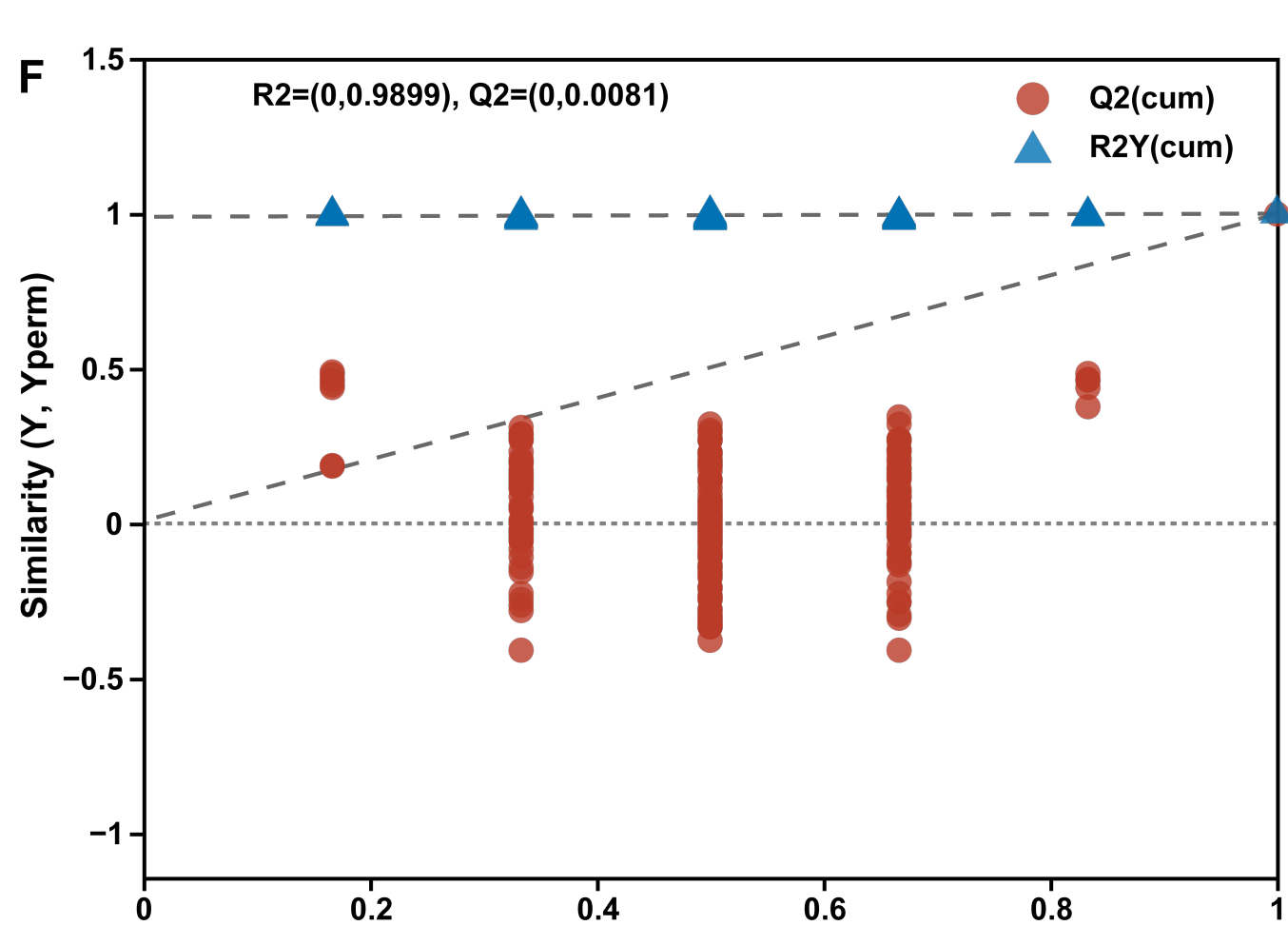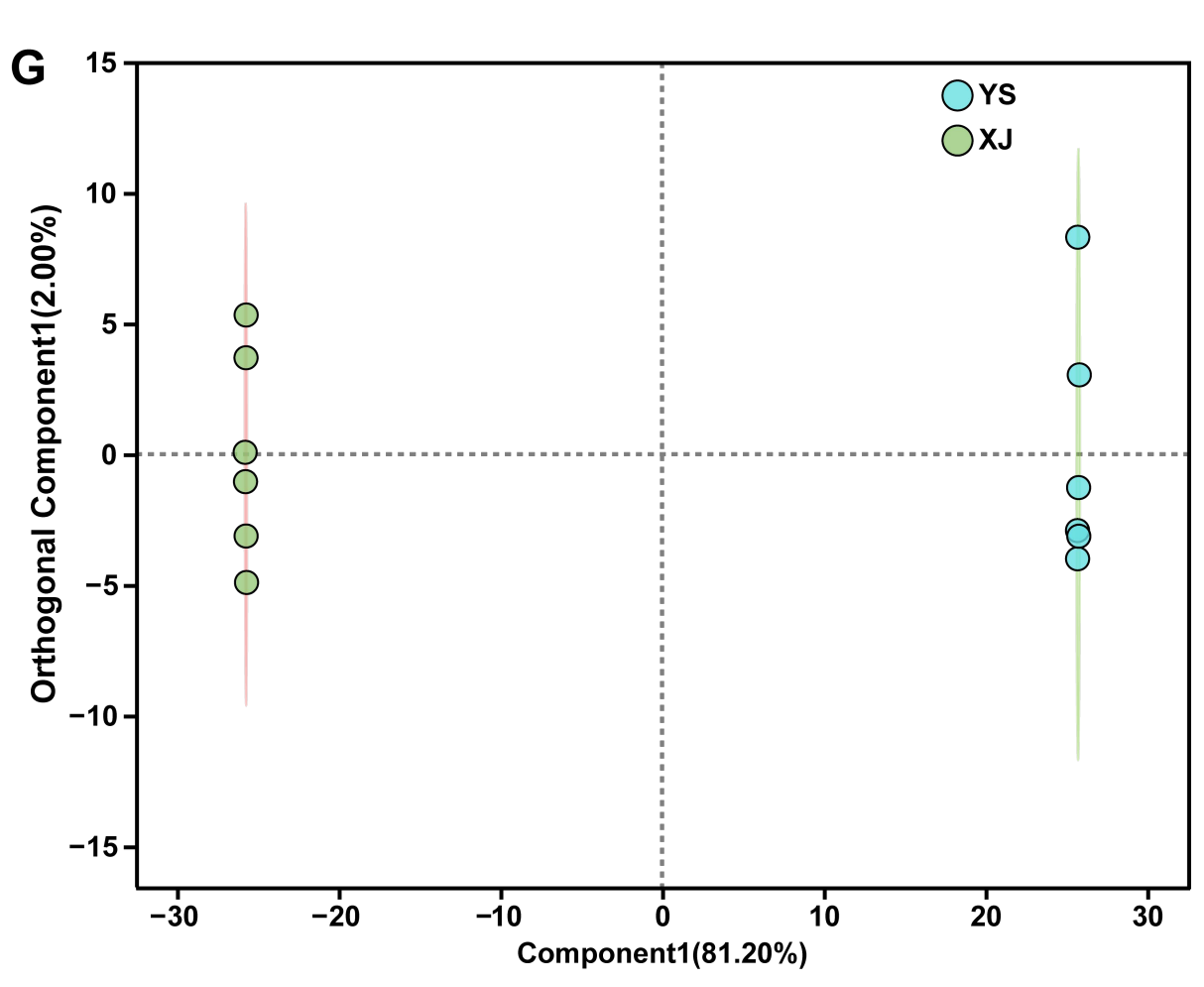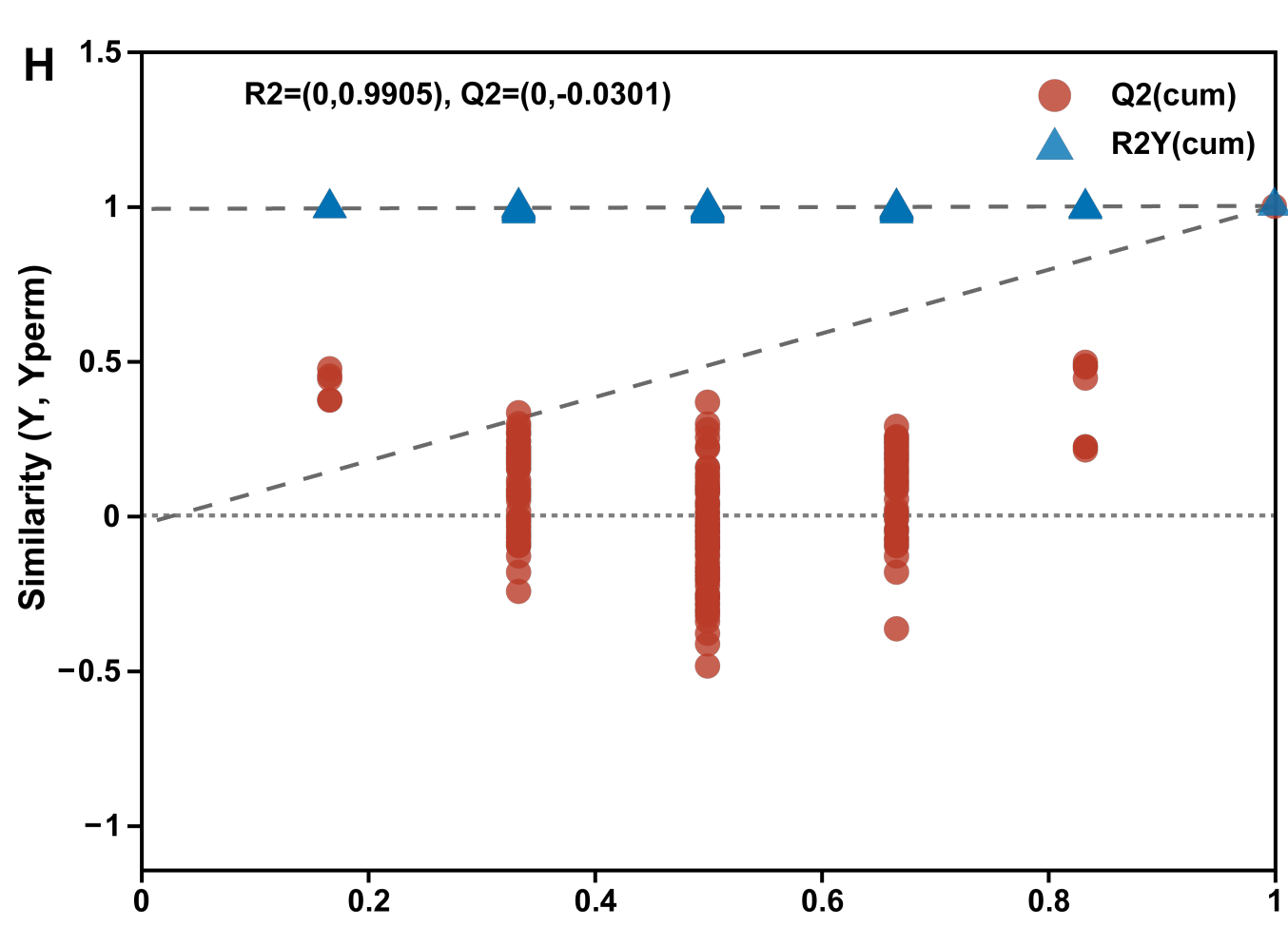

Supplement: Supplementary file 1 [file metabolites-14-00414-s001.zip › metabolites-3093316-supplementary-Figure S1.pdf]
